# Monitoring of Venus transgenic cell migration during pregnancy in non-transgenic rabbits

**DOI:** 10.1007/s11248-016-9994-9

**Published:** 2016-11-10

**Authors:** N. Lipták, O. I. Hoffmann, A. Kerekes, G. Iski, D. Ernszt, K. Kvell, L. Hiripi, Z. Bősze

**Affiliations:** 1 NARIC-Agricultural Biotechnology Institute, Gödöllő, Hungary; 2 Department of Pharmaceutical Biotechnology, University of Pécs, Pécs, Hungary

**Keywords:** Microchimerism, CAGGS promoter, Sleeping Beauty transposition, Non-transgenic, New Zealand white rabbits, Venus protein

## Abstract

Cell transfer between mother and fetus were demonstrated previously in several species which possess haemochorial placenta (e.g. in humans, mice, rats, etc.). Here we report the assessment of fetal and maternal microchimerism in non-transgenic (non-TG) New Zealand white rabbits which were pregnant with transgenic (TG) fetuses and in non-TG newborns of TG does. The TG construct, including the Venus fluorophore cDNA driven by a ubiquitous cytomegalovirus enhancer, chicken ß-actin promoter (CAGGS), was previously integrated into the rabbit genome by Sleeping Beauty transposon system. Three different methods [fluorescence microscopy, flow cytometry and quantitative polymerase chain reaction (QPCR)] were employed to search for TG cells and gene products in blood and other tissues of non-TG rabbits. Venus positive peripheral blood mononuclear cells (PBMCs) were not detected in the blood of non-TG littermates or non-TG does by flow cytometry. Tissue samples (liver, kidney, skeletal and heart muscle) also proved to be Venus negative examined with fluorescence microscopy, while histology sections and PBMCs of TG rabbits showed robust Venus protein expression. In case of genomic DNA (gDNA) sourced from tissue samples of non-TG rabbits, CAGGS promoter-specific fragments could not be amplified by QPCR. Our data showed the lack of detectable cell transfer between TG and non-TG rabbits during gestation.

**Electronic supplementary material:**

The online version of this article (doi:10.1007/s11248-016-9994-9) contains supplementary material, which is available to authorized users.

## Introduction

TG rabbits are widely used in biomedical research and great tools for studying cardiovascular failures, e.g. hypertrophic cardiomyopathy (Marian et al. 1999), cardiac arrhythmias (Brunner et al. 2008; Major et al. 2016); etc. In addition, TG animals, especially rabbits, are also capable to secrete proteins as bioreactors (Bijvoet et al. 1999; Bodrogi et al. 2006; Hiripi et al. 2003; Hoeg et al. 1996).

In spite of these improvements in research, development of TG animals for food supply is still not permitted in the vast majority of countries. There is only one GM animal; the AquAdvantage Salmon which was approved by United States Food and Drug Administration to commercialize for food production in 2015. Due to the strict legal regulations, it is essential to investigate the presence of TG cells and/or transgene products in livestock animals. If TG animals have non-TG offspring or a non-TG mother is pregnant with TG littermates, the transfer of transgenic DNAs and/or cells between mother and fetuses or between fetuses during pregnancy is possible. These DNA and/or cell migrations are called fetal or maternal microchimerism, which are occurring during normal gestation (Gammill and Nelson 2010).

Fetal cell microchimerism was firstly identified in pregnant women (Schroder and De la Chapelle 1972; Walknowska et al. 1969). Fetal cell microchimerism was also discovered in animals with similar haemochorial placentation as human: in mice (Matsubara et al. 2009; Zhang et al. 2014), rats (Wang et al. 2004) (haemotrichorial placenta), and rhesus monkeys (Jimenez et al. 2005) (haemomonochorial placenta). These cells were observed in blood and other tissues (e.g. kidney, spinal cord, liver, etc.). The haemochorial placenta has invasive trophoblasts that support direct contact to the maternal circulation. In contrast, fetal microchimerism in livestock animals with epitheliochorial placentation is usually not detectable (Garrels et al. 2014; Steinkraus et al. 2012), and only demonstrated in cattle (Pereira et al. 2013). Epitheliochorial placenta contains multiple layers which separate the maternal and fetal blood vessels. Therefore, epitheliochorial placentation is more effective in blocking cell migration between mother and fetuses during pregnancy than haemochorial placenta.

Rabbits (*Oryctolagus cuniculus*) are used as domestic animals for human consumption and laboratory animals for scientific research as well. Rabbits have haemodichorial placenta (Enders 1965) with two cellular layers of chorion between the fetal and maternal blood.

In this study we examined the fetal–maternal, maternal–fetal and fetal–fetal cell microchimerism in transgenic rabbits expressing the Venus fluorophore reporter protein. Venus construct was integrated into the rabbit genome using Sleeping Beauty transposon system (Katter et al. 2013). This system contains the hyperactive SB100X transposase (pCMV-SB100X) and the transposon donor plasmid, which carries a Venus fluorophore driven by the CAGGS promoter (Mates et al. 2009). Venus protein is a yellow shifted variant (excitation maximum at 515 nm) of the widely used enhanced green fluorescent protein (EGFP, excitation maximum at 488 nm). Venus protein was expressed in all examined organs and tissues in TG rabbits (Katter et al. 2013). Therefore, this expression pattern gave us a great opportunity to search for Venus positive cells in the blood and other tissues of wild type does and newborn rabbits.

## Materials and methods

### Animals

Wild type and SB-CAGGS-Venus TG New Zealand White rabbits at age of 5–10 months were used in this study. All TG animal used in this study were heterozygote for the transgene. Animals were kept under a standard light–dark cycle (lights on between 07.00 and 19.00 h) at 18 ± 3 °C with food and water available ad libitum and caged separately. All experiments were approved by the Animal Care and Ethics Committee of the NARIC-Agricultural Biotechnology Center (Gödöllő, Hungary) and the Pest County’s governmental office (permission numbers: PEI/001/329-4/2013; PEI/01/857-3/2015). Animals were kept and treated according to the rules of the Hungarian Code of Practice for the Care and Use of Animals for Scientific Purposes.

### Semen collection and artificial insemination

Semen was collected from both TG bucks carrying one monomeric integration of the SB-CAGGS Venus transgene and from non-TG bucks using artificial vagina (Morrell 1995).

Ten does were injected with 0.2 mL GnRH analog intramuscularly (Receptal, Intervet International B.V. Boxmeer) to induce ovulation. Five out of ten does were inseminated with sperm from TG bucks to investigate cell trafficking between fetuses and between does and their fetuses. For detection of the maternal–fetal microchimerism, three TG does were fertilized with sperm from non-TG bucks. Other two non-TG does were fertilized with non-TG sperm as negative controls.

### Blood collection protocol

Peripheral blood samples (2 mL) were collected into EDTA containing tubes from the ear arteries of does before insemination; on the 10th, 20th days of pregnancy; before parturition and after they gave birth. In addition, blood samples were also obtained from the bucks used for insemination (ID numbers: #4016, #4031) and from a non-TG control. After birth, newborn rabbits were sacrificed and blood samples were withdrawn (2 mL) from common carotid arteries (7 from non-TG♂ × non-TG♀; 15 from TG♂ × non-TG♀ and 11 from non-TG♂ × TG♀ crossings). Furthermore, blood samples were obtained from six adult TG rabbits (ID numbers: #4016, #4031, #4032, #4033, #4034 and #4036; all from heterozygote × heterozygote crossings; 4th generation of the transgenic line).

### Isolation of PBMCs

Peripheral blood was centrifuged at 800 g for 20 min at room temperature using Lymphoprep (Axis-Shield) density gradient media to isolate PBMCs. PBMC samples were fixed in 0.5 mL 0.5% (w/v) paraformaldehyde (PFA) for flow cytometry analysis. Venus positive PBMCs in peripheral blood of pregnant non-TG does and non-TG newborns were considered as foreign TG cells.

### Selection of TG and non-TG newborn rabbits

As we described previously (Katter et al. 2013), the fluorescence of Venus protein was detected using blue light illumination (FSH/LS-1B) with a barrier filter cutoff below 500 nm with a GFSP-5 headset (BLS, Hungary). Newborns with macroscopic excitation of Venus fluorescence protein were identified as TG rabbits and also confirmed as TG by PCR.

### Detection of TG cells by flow cytometry analysis

The Venus positivity of PBMC samples were analysed with FACSCanto II flow cytometer (Becton–Dickinson). PBMCs were gated in FSC/SSC plot and the percentage of Venus positive cells was determined by the signal of FITC channel. Data were acquired and analysed with FACSDiva software (Becton–Dickinson). Approximately 14–15.000 PBMCs were measured from each sample.

### Histology and fluorescence microscopy

Tissue samples from non-TG does, non-TG newborns and two TG newborns (liver, kidney, heart, skeletal muscle) were removed after birth, washed in PBS and fixed in 4% (w/v) PFA at 4 °C for 2 days. After incubation in PFA, samples were replaced in ascending concentration (10, 20, 30 w/v%) of sucrose-PBS solution. Samples were stored in each concentration of sucrose-PBS solution for 24 h at 4 °C, respectively. Samples were incubated in 30% sucrose-PBS solution until embedding.

Tissue samples were embedded into cryomedium before cryosectioning (Cryomatrix, Thermo Scientific) and cut into 10 µm thick sections on a cryostat (Microm, Heidelberg). Ten sections per organ were prepared from each tissue. Sections were mounted with FluorSave reagent (Merck Millipore), coverslipped, and observed under a fluorescence microscope with appropriate filters. Figures were created in GIMP 2.8.14 graphics program.

### Quantitative polymerase chain reaction (QPCR)

gDNA was purified from liver, skeletal muscle, kidney and heart samples using the proteinase K method. QPCR was performed using Power SYBR Green PCR mix (Applied Biosystems, Life Technologies) with a real time rotary analyzer (Rotor-Gene 3000, Corbett Life Science). The same primer pair was used for amplification of the CAGGS promoter fragments as published in a previous study (Garrels et al. 2014).

### CAGGS promoter specific primer pairs

Reverse: 5′-GCAGCCACAGAAAAGAAACGA- 3′

Forward: 5′-GCTCTGACTGACCGCGTTACT- 3′

The PCR mix contained 10 µl Power SYBR Green PCR mix, 7 µl dH_2_O, 1 µl of reverse and forward primer pairs (5 µM) and 1 µl of gDNA (50 ng) in 20 µl total volume. Concentration of gDNA samples were verified with the ND-1000 NanoDrop spectrophotometer (Thermo Scientific). Taq polymerase was activated at 95 °C for 10 min followed by 45 cycles of denaturation at 95 °C for 15 s, and annealing at 61 °C for 1 min. Reactions were performed in triplicates of each gDNA samples. #4033 CAGGS-Venus transgenic doe and a TG littermate (#4036.4) were used as positive controls. Non-TG littermates from non-TG♂ × non-TG♀ crossing (#3.1, #3.2, and #3.3) were taken as negative controls. In addition, water (no- template DNA) controls were also added in triplicates to all real-time reactions.

### Statistical analysis

Statistical analysis of the flow cytometry and QPCR data were made by one way analysis of variance (ANOVA). A probability value, *P* < 0.05 was considered statistically significant.

## Results and discussion

Investigation of the possible presence of TG cells or gene products in domestic animals is important due to the unpredictable impacts of TG animals on food chain. Fetal and maternal microchimerism is a potential source of TG cells in non-TG animals. Microchimerism defined as the occurrence of small number of foreign cells (less than 1:100 cells) in the host animal. Pregnancy-related microchimerism was already examined in livestock EGFP TG animals [pig (Garrels et al. 2014), cattle (Pereira et al. 2013)] by reason of the easy detection of the fluorophore protein in PBMCs and tissue samples. Although rabbit meat consumption is still global [for details, see review (Zotte 2014)], the migration of TG cells during pregnancy in rabbits is still an opened question.

**Fig. 1:**
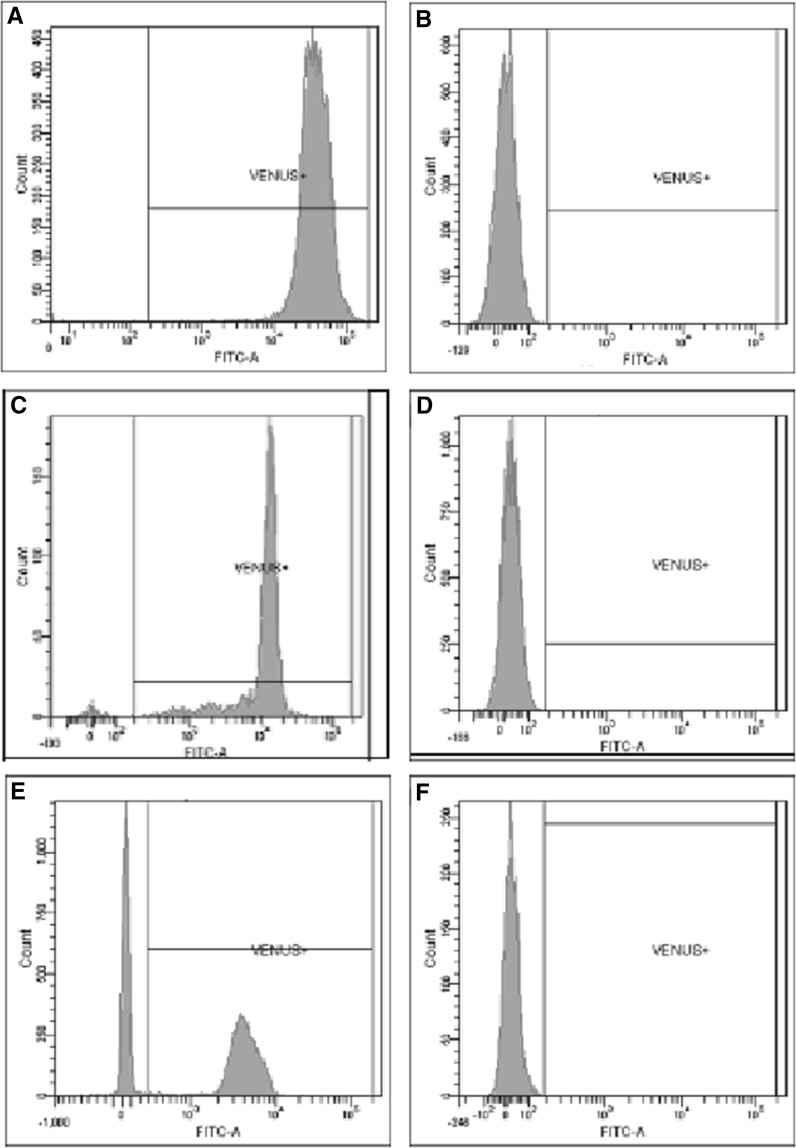
The detection of TG cells by flow cytometry. **A** SB-CAGGS-Venus TG buck (#4016); **B** non-TG buck; **C** SB-CAGGS-Venus TG doe (#4036); **D** #2 doe, at 20th day of pregnancy; **E** SB-CAGGS-Venus TG newborn #5.2.; **F** non-TG newborn #1.1

Flow cytometry, QPCR and tissue histology are suitable methods for detection of microchimerism. The matings and the number of newborns used in this study are presented in Table 1. Almost all PBMCs of the TG bucks (99.9% in #4016 and #4031) used for insemination proved to be Venus positive (Fig. 1a), while blood sample of non-TG buck was Venus negative (Fig. 1b). Nearly all PBMCs of the adult SB-CAGGS-Venus TG rabbits were Venus positive, including TG does (Fig. 1c) which carried non-TG and TG fetuses (see statistics in Table 2). There were no detectable Venus PBMCs in non-TG does inseminated with TG sperm before insemination and at any time points (Fig. 1d). Both Venus negative and positive PBMCs were found in blood of TG newborns (Fig. 1e). Our flow cytometry results showed the absence of Venus positive cells in PBMCs of the non-TG littermates from TG♂ × non-TG♀ (n = 9, Fig. 1f) and non-TG♂ × TG♀ crossings (n = 6).

**Table 1:** The number of non-TG and TG newborns after artificial insemination

Buck genotype	Does genotype	Litter size	Number of TG newborns
SB-CAGGS-Venus #4016	#1 non-TG control	8	4
	#2 non-TG control	7	3
	#5 non-TG control	4	3
SB-CAGGS-Venus #4031	#10 non-TG control	6	5
Non-TG control	SB-CAGGS-Venus #4034	5	4
	SB- CAGGS-Venus #4036	8	5
	SB-CAGGS-Venus #4033	8	5
Non-TG control	#3 non-TG control	7	0
	#6 non-TG control	5	0

The size of litters was similar in all type of crossing. Lower fertility was not observed in case of non-TG♂ × TG♀ and TG♂ × non-TG♀ matings. Newborns were named with the ID number of the does followed by a number reflecting the order of blood collection. For example, #5 doe had four newborns, one non-TG (#5.1) and three TG (#5.2–#5.4)

**Table 2:** The absence of detectable PBMC transfer during pregnancy

Group	1	2	3	4	5	6
	TG newborns (TG♂ × non-TG♀) (n = 6)	Adult TG rabbits (TG♂ × TG♀) (n = 6)	Non-TG newborns (non-TG♂ × non-TG♂♀) (n = 7)	TG newborns (non-TG♂ × TG♀) (n = 5)	Non-TG newborns (non-TG♂ × TG♀) (n = 6)	Non-TG newborns (TG♂ × non-TG♀) (n = 9)
Venus positive PBMCs (%)	71.1 ± 14.2	97.2 ± 1.4*	0.0 ± 0.0	84.0 ± 7.9	0.0 ± 0.0	0.0 ± 0.0

Percentages are represented in mean ± S.D

* *P* < 0.001 compared to Group 1 and Group 4, F_(5,33)_ = 363.87 (one way ANOVA, Scheffe post hoc)

Numbers in brackets indicate the crossings and the numbers of rabbits used in these experiments. Venus positive PBMCs were not detected in the blood of non-TG newborns and non-TG does (n = 3) in any crossings

**Fig. 2:**
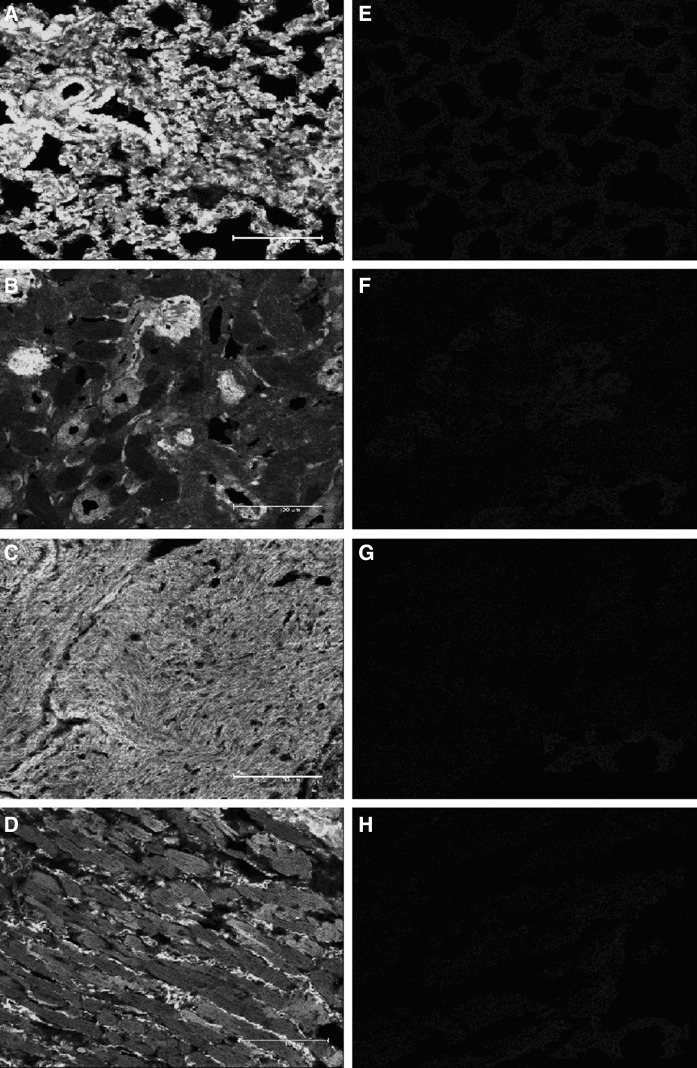
The expression of Venus fluorescence protein in selected organs of newborns. Tissue sections of #5.2 SB-CAGGS-Venus TG newborn were Venus positive (**A** liver, **B** kidney, **C** heart, **D** skeletal muscle). Sections of #1.1 non-TG newborn did not express Venus fluorescence protein (**E** liver, **F** kidney, **G** heart, **H** skeletal muscle). The organs of other non-TG newborns and non-TG does also remained Venus negative (sections not shown). *Scale bars* (*bottom right*) in all images are 100 µm. Images were captured on a Leica SP5 confocal laser scanning microscope (Leica Microsystems) at ×400 magnification

Supporting these observations, Venus positive cells were also not found in the tissue sections of these non-TG newborn rabbits (Fig. 2e–h) while TG newborns showed strong Venus specific fluorescence (Fig. 2a–d), similarly to adult TG rabbits (Katter et al. 2013). Additionally, Venus positive cells were detected in non-TG does delivered TG newborns neither by flow cytometry (Fig. 1d) nor by fluorescence microscopy. Notably, significant difference was also observed in the percentage of Venus positive cells between adult and newborn TG rabbits by flow cytometry (Table 2), suggesting the age-dependent expression of Venus protein in blood PBMCs.

In QPCR experiments, to specify the detection limit, a dilution series of SB-CAGGS Venus gDNA was created. SB-CAGGS Venus gDNA samples were mixed with wild type rabbit gDNA to simulate one positive cell in approximately 100, 1000 and 10,000 cells. Our detection limit was one Venus positive TG cell in 1000 cells (see details in Table 3). Ct values of QPCR reactions contained 50 ng/µl SB-CAGGS Venus gDNA were always under 24.5. Non-TG control littermates from both non-TG♂ × TG♀ and TG♂ × non-TG♀ crossings had Ct values above 29.8 so did the non-TG does (see statistics in supplementary Table 1). Therefore, QPCR reactions above 29 threshold cycles considered lacking of CAGGS promoter specific products. We could not detect any microchimeric cell in non-TG offspring and non-TG does from any crossings. The amplified fragment from a TG newborn was sequenced and analyzed. Analysis showed 100% identity to CAGGS of our injected plasmid used for transgenesis (data not shown).

**Table 3:** The detection limit of the CAGGS-specific fragments in QPCR experiments

	TG rabbit (only Venus positive cells)	Venus positive cells (1:100 dilution)	Venus positive cells (1:1000 dilution)	Venus positive cells (1:10,000 dilution)	Non-TG does and non-TG offspring from any crossings
QPCR	+	+	+	−	−

Overall, these data indicated there was no detectable cell transfer in utero between TG and non-TG in rabbits despite the haemochorial placentation. In an earlier study, GFP positive fetal cells were detected in maternal peripheral blood during pregnancy and postpartum in non-TG female mice which were mated with GFP TG mice (Matsubara et al. 2009). Maternal PBMCs were also observed in peripheral blood of the offspring (Vernochet et al. 2005) and maternal T cells were detected in fetal blood (Nijagal et al. 2011) in mice. In contrast to rabbits, maternal–fetal cell traffic is well described in mice from prenatal period to adulthood in various tissues (for more data, see review (Stelzer et al. 2015)). Moreover, the smaller total blood volume and the size of the examined organs in mice compared with rabbits could facilitate to find microchimeric cells. In the present study, 2 mL blood were obtained from each pregnant does at three different time points during pregnancy and once after they gave birth. This blood volume is approximately 1–2% of the total blood volume of an adult rabbit. In pregnant women, the collection of similar percentage of the total blood volume was sufficient to detect fetal cells (Evans et al. 1999; Schroder and De la Chapelle 1972). The available data in literature about fetal microchimerism within same species, especially in women, are also contradictory. Numerous publications provided data about fetal microchimerism after pregnancy in women [for details, see review (Gammill and Nelson 2010)], but studies claiming no evidence for fetal DNA in maternal plasma after delivery were also published (Lo et al. 1999; Smid et al. 2003). Our results based on three different methods suggest that beside the prominent role of placentation, the influence of species-specific differences in pregnancy-related microchimerism cannot be ruled out.

QPCR is a generally accepted method not only for detection of microchimerism, but for quantification of GMO in the meat of non-TG domestic animals. The vast majority of studies published data about detection of plant GMO contamination in meat. The presence of Roundup Ready GMO soybean in commercially available processed meat was identified in Brazil (Dinon et al. 2010), Serbia (Taski-Ajdukovic et al. 2009), Hungary (Ujhelyi et al. 2008), etc. In these publications there were GMO positive samples which exceeded the threshold of label legislation for GMO contamination (1% in Brazil and 0.9% in the European Union). In case of CAGGS promoter specific amplification, our QPCR data showed significantly lower Ct values at 1:100 dilution of Venus genomic DNA compared to non-TG gDNA (see details in Table 3 and supplementary Table 1). Thus, these non-TG rabbits which were bred only for experimental purposes did not contain GMO above the threshold of label legislation for food products.

## Electronic supplementary material

Below is the link to the electronic supplementary material.

Supplementary material 1 (DOCX 12 kb)
