# Supplementary material for: Monitoring of Venus transgenic cell migration during pregnancy in non-transgenic rabbits
Source: Transgenic Res. 2016 Nov 10;26(2):291–9. doi: 10.1007/s11248-016-9994-9 (PMC5350230; doi:10.1007/s11248-016-9994-9)
Supplement: Supplementary file 1 — Supplementary material 1 (DOCX 12 kb) [file 11248_2016_9994_MOESM1_ESM.docx]

**Supplementary Table 1: No measurable CAGGS promoter fragments in gDNA of non-TG newborns and does in QPCR**

| **Panel A: Purified gDNA from newborns** | | | |
| --- | --- | --- | --- |
| **Examined groups** | | **Ct values (**mean ± S.D.) | |
|  |  | **Muscle** | **Liver** |
| Dilution of Venus gDNA (ng/ µl)  (TG doe) | 50 | 21.60±0.30 | 22.07±0.12 |
|  | 0.5 | 26.90±0.46 | 26.57±0.35 |
|  | 0.05 | 29.37±0.15 | 29.73±0.68 |
|  | 0.005 | 29.47±0.38 | 29.73±0.31 |
| non-TG littermates (non-TG♂ x non-TG♀ crossing, #3.1, #3.2, #3.3) | | 29.80±0.39* | 30.11±0.43** |
| TG littermate (non-TG♂ x TG♀ crossing, #4036.4) | | 23.97±0.06 | 22.57±0.21 |
| non-TG littermates (non-TG♂ x TG♀ crossing, #4033.2, #4036.1, #4036.2) | | 29.86±0.43* | 30.29±0.28** |
|  | | | |
| **Panel B: Purified gDNA from newborns and does** | | | |
| **Examined groups** | | **Ct values (**mean ± S.D.) | |
|  |  | **Muscle** | **Liver** |
| Dilution of Venus gDNA (ng/ µl)  TG doe (#4033) | 50 | 22.50±0.82 | 22.13±0.76 |
|  | 0.5 | 27.70±0.79 | 26.93±0.40 |
|  | 0.05 | 31.17±2.51 | 32.00±1.30 |
|  | 0.005 | 33.75±0.21 | 32.85±0.49 |
| non-TG littermates (non-TG♂ x non-TG♀ crossing, #3.1, #3.2, #3.3) | | 35.35±2.25* | 34.39±1.92** |
| TG littermate (non-TG♂ x TG♀ crossing, #4036.4) | | 23.95±0.78 | 22.97±0.42 |
| Non-TG does carrying TG fetuses  (#1, #2, #5 does) | | 34.40±2.16* | 33.56±0.38** |

Asterisks represent significant differences between groups (one way ANOVA, Scheffe post-hoc).

**Panel A**

Non-TG littermates (both from non-TG♂ x non-TG♀ and non-TG♂ x TG♀ crossings) had significantly higher mean Ct values compared to TG littermate and TG doe (50 ng/µl and 0.5 ng/µl Venus gDNA)

* muscle samples of non-TG newborns: F_(6,24)_ = 290.12, p < 0.001;

** liver samples of non-TG newborns: F_(6,26)_ = 367.98, p < 0.001;

**Panel B**

Non-TG littermates (from non-TG♂ x non-TG♀ crossing) and non-TG does carrying TG fetuses also significantly higher mean Ct values compared to TG littermate and TG doe (50 ng/µl and 0.5 ng/µl Venus gDNA):

* muscle samples of non-TG newborns and non-TG does: F_(6,20)_ = 25.55, p < 0.001

** liver samples of non-TG newborns and non-TG does: F_(6,23)_ = 74.79, p < 0.001

In case of kidney and heart tissues, Ct values were similar (in TG rabbits: under 23, non-TG rabbits: above 30) as in real-time reactions including muscle and liver gDNA samples.
